# ISSR and AFLP analysis of the temporal and spatial population structure of the post-fire annual, *Nicotiana attenuata*, in SW Utah

**DOI:** 10.1186/1472-6785-4-12

**Published:** 2004-09-06

**Authors:** Rahul A Bahulikar, Dominic Stanculescu, Catherine A Preston, Ian T Baldwin

**Affiliations:** 1 Max Planck Institute for Chemical Ecology, Dept of Molecular Ecology, Hans Knöll Strasse 8, Beutenberg Campus, 07745 Jena Germany; 2 Fachbereich Biologie, Universität Konstanz, 78457 Konstanz, Germany; 3 USDA-ARS CMAVE, 1600/1700 SW 23^rd ^Drive, Gainesville, FL 32608 USA

## Abstract

**Background:**

The native annual tobacco, *Nicotiana attenuata*, is found primarily in large ephemeral populations (typically for less than 3 growing seasons) after fires in sagebrush and pinyon-juniper ecosystems and in small persistent populations (for many growing seasons) in isolated washes typically along roadsides throughout the Great Basin Desert of the SW USA. This distribution pattern is due to its unusual germination behavior. Ephemeral populations are produced by the germination of dormant seeds from long-lived seed banks which are stimulated to germinate by a combination of unidentified positive cues found in wood smoke and the removal of inhibitors leached from the unburned litter of the dominant vegetation. Persistent populations may result where these inhibitors do not exist, as in washes or along disturbed roadsides. To determine if this germination behavior has influenced population structure, we conducted an AFLP (244 individuals), ISSR (175 individuals) and ISSR+ AFLP (175 individuals) analysis on plants originating from seed collected from populations growing in 11 wash and burns over 11 years from the SW USA.

**Results:**

Genetic variance as measured by both ISSR and AFLP markers was low among sites and comparatively higher within populations. Cluster analysis of the Utah samples with samples collected from Arizona, California, and Oregon as out-groups also did not reveal patterns. AMOVA analysis of the combined AFLP and ISSR data sets yielded significantly low genetic differentiation among sites (Φct), moderate among populations within sites (Φsc) and higher genetic differentiation within populations (Φst).

**Conclusions:**

We conclude that the seed dormancy of this post-fire annual and its resulting age structure in conjunction with natural selection processes are responsible for significantly low among sites and comparatively high within-population genetic variation observed in this species.

## Background

*Nicotiana attenuata *Torr. ex Watson (Solanaceae) (synonymous with *N. torreyana *Nelson and Macbr.) is an annual native to the Great Basin Desert of California, Nevada, Idaho, and Utah (USA) [1-3] and primarily occurs in large ephemeral populations (typically for less than 3 growing seasons) after fire in sagebrush and pinyon-juniper ecosystems, in small persistent (for >3 growing seasons) populations in isolated washes, and as a roadside weed after new construction in a previously undisturbed area [2,4-9]. Positive and negative control by environmental signals over germination from long-lived seed banks (estimated to be minimally 150 y [10] can account for its occurrence in these habitats. Specifically, dormant *N. attenuata *seeds are stimulated to germinate by unidentified factors in wood smoke [9] but are inhibited by factors, including ABA and 4 terpenes (bornane-2,5-dione, 1,8-cineole, β-thujaplicin and camphor [11] which leach from the litter of the dominant vegetation. Genotypes of *N. attenuata *produce seeds that vary in their genetically-determined primary dormancy [9]. Regardless of their degree of primary dormancy, seeds that are shed in unburned habitats with significant accumulations of litter develop strong secondary dormancy in response to the negative germination cues. If the seeds are shed into habitats without significant litter accumulations (e.g. in washes or roadside habitats), seeds without dormancy germinate. When fires pyrolyze the litter layer, removing the germination inhibitors and saturating the soils with smoke-derived germination stimulants, the seed bank responds with a dramatic, synchronized germination response the following growing season during favorable moisture and thermal regimes.

This well-characterized germination behavior likely affects the genetic structure of this potential annual. Genetic structure of a population results from mutations, gene flow (as mediated by pollen and seed dispersal), drift, and selection, all acting in the context of an organism's life history traits [12]. Genetic differentiation may be more prevalent between primarily dormant and non-dormant populations, namely between plants found ephemerally (in burns) and those occurring more persistently (in washes). Within the ephemeral populations, the number of plants in the population will vary in relation to the size of the burn and the distribution of the seed bank. Because pollinators must locate these ephemeral populations in a landscape that may be largely composed of other plant associations, out-crossing may not be prevalent. Flowers of *N. attenuata *are self-compatible and outcrossing does not significantly affect seed production, seed mass or viability [13] indicating that this species relies on selfing as its primary form of reproduction. Selfing may keep genetic variation low, especially within populations. Persistent populations are more likely to experience outcrossing, owing to their predictability. These considerations in combination with the annual life cycle of plants in washes in contrast to the 7 – 150 year life cycle of plants growing in burns may increase the genetic differences among populations found in burns and washes.

**Figure 1:**
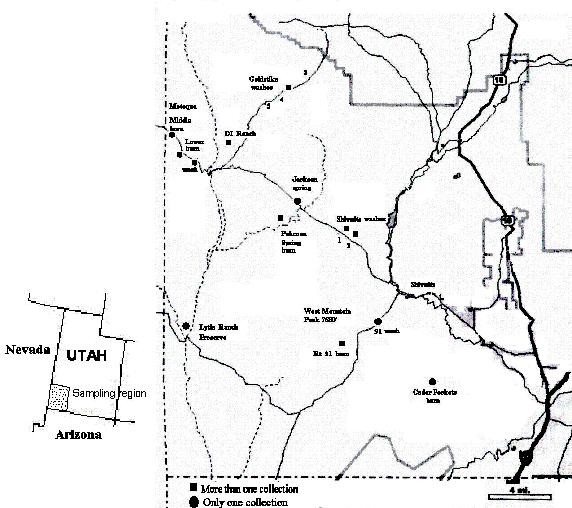
**Location of *Nicotiana attenuata *populations from which seeds were collected between 1988–1999 in Southwestern Utah**. See Table 1 for number of plants grown for DNA extraction from each location for the AFLP and ISSR analysis. Locations labeled with circles represent single-site or -time collections, while squares signify multiple-site or -time collections

Here we examine the genetic structure of *N. attenuata *plants from wash and burn populations in the SW Utah (Fig. 1; Table 1) to determine if the particular germination behavior of this species has left signatures in the plant's population structure. We use an AFLP (amplified fragment length polymorphism) analysis, based on the selective polymerase chain reaction (PCR) amplification of restriction fragments from a total digest of genomic DNA [14] and an ISSR (inter-simple sequence repeats) analysis in which bands are generated by single primer PCR that amplifies products between two simple sequence repeats [15]. Both procedures produce reproducible markers useful for the quantification of genetic polymorphism within species [16].

**Table 1:** Number of individual-plant DNA samples harvested for AFLP and ISSR analysis of plants grown from seed collected from: A) six sites within Utah collected over multiple years; B) three burns and 4 roadside washes within Utah collected in 1999; C) 3 non-Utah collections.(Codes identify samples in Fig. 3 and Table 5 [see Additional file 1]

**Location**	**Seed collection Year**	**Codes**	**SET I Used for only AFLP**	**SET II Used for ISSR & AFLP**	**Burn(B) or Wash (W)**
**A. Time series from the following Utah sites:1**					
1. Motoqua roadside wash and burn (fire in 1994)					
Wash	1990	M1	4		W
Lower Burn	1995	M2	3		B
Middle burn	1995	M3	3		B
Middle burn	1996	M4	8	4	B
Lower Burn	1996	M5	3	3	B
Wash	1999	M6	4	8	W
2. DI Ranch burns (yearly fires at garbage dump)					
	1988	D1	4		B
	1990	D2	1		B
	1992	D3	6		B
	1993	D4	2		B
	1995	D5	4	4	B
3. Goldstrike roadside washes					
#2	1990		2		W
#5	1990		1		W
#2	1993	G1	5	5	W
#4	1993	G2	2	2	W
#5	1993	G3	1	1	W
4. Shivwits Reservation roadside wash # 1					
	1988		1		W
	1990		2		W
	1992		1		W
	1993		4		W
	1995		1		W
	1999	B7	8	8	W
5. Shivwits Reservation roadside wash # 2					
	1992	B1	1		W
	1993	B2	3		W
	1995	B3	4		W
	1996	B4	5		W
	1999	B5	9	8	W
	1999	B6	1		W
6. Pahcoon Spring roadside wash					
	1990		1		W
	1999	P9	9	8	W
**B) Burn and roadside wash populations collected in Utah 1999**					
1. Pahcoon Spring Burn (fire in 1998)					
Burn transect 1	1999	P1	9	6	B
Burn transect 2	1999	P2	7	5	B
Burn transect 3	1999	P3	9	8	B
Burn transect 4	1999	P4	10	8	B
Burn area 1	1999	P5	9	8	B
Burn area 2	1999	P6	9	8	B
Burn area 3	1999	P7	9	7	B
Burn area 4	1999	P8	8	8	B
2. Rt 91 Burn (fire in 1998)					
Burn area 1	1999	R1	8	6	B
Burn area 2	1999	R2	8	8	B
3. Single collection roadside washes and burns					
Shivwits Reservation 4	1999	B8	9	8	W
Rt-91	1999	R3	9	7	W
Lytle Ranch Preserve	1999	L1	10	10	W
Jackson Spring	1999	J1	10	10	W
Cedar Pockets	1999	C1	10	10	B
C) Non-Utah collections					
Arizona	1996		3	3	
Oregon	1994		2	2	
California	1999		2	2	
Total plants			244	175	

Specifically, we compare plants growing from seeds collected from 11 large populations after fires, from small populations in 10 washes, from plants in transects across 5 large burns, and from plants growing in specific areas over 10 years during which a small wash population erupted into a large burn population as a result of a fire and returned to become a small wash population. By analyzing the genetic diversity across these *N. attenuata *populations, we aimed to answer the following questions: 1) Are plants growing in burn and wash populations genetically distinct? 2) Are plants growing in the same washes genetically similar through different years? 3) What is the genetic makeup of plants found growing across large burns and geographically adjacent populations? While genetic diversity among the various *Nicotiana *species has been studied with RAPD [17] and AFLP [18] markers, and with peroxidase isozymes [19], this is the first effort to study the spatial and temporal population structure of a native *Nicotiana *species.

## Results

### SET-I

**Figure 2:**
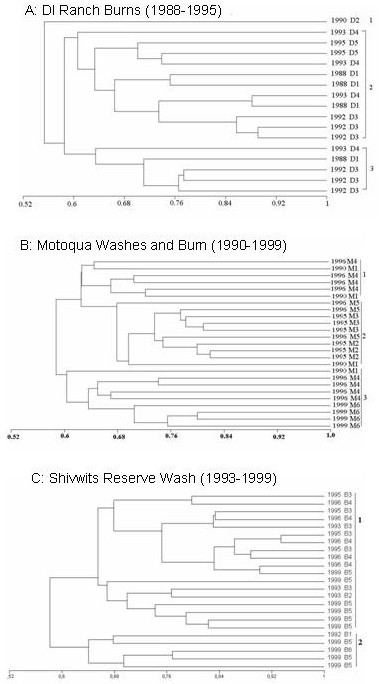
**UPGMA dendrograms based on the Jaccard similarity index calculated from an AFLP analysis of *N. attenuata *populations collected at three different sites. **A: DI Ranch (17 individuals); B: Motoqua Burn and Wash (25 individuals); C: Shivwits Reservation (23 individuals) collected over a number of different years. Sample codes are given in Table 1. While substantial genetic variation was found, this variation was not organized in time.

Set I (Table 1) consisting of 244 individuals, which was used only for AFLP analysis, produced a total of 207 loci (data not shown). This data was used for separate dendrogram and principle co-ordinate (PCO) analyses. The Jaccard similarity index [20] based on unweighted pair group method average (UPGMA) dendrogram revealed a lack of distinct spatial or temporal structure and had brush- or star-structures with nodal bootstrap values of less than 60% (data not shown). The samples collected from the greatest spatial distances, namely California, Oregon and Arizona, did not form separate clusters from any of the Utah populations. A cluster analysis of 10 wash and 5 burn populations all grown from seed collected in 1999 (Table 1) revealed no clustering based either on type of population (wash or burn) or geographic location (data not shown).. Some structure was identified when each time series at particular locations (Motoqua, DI Ranch, Shivwits Reservation) were analyzed separately (Fig. 2A,2B,2C), but the nodes did not correspond to a particular growing season. No genetic differentiation was observed from a cluster analysis of plants collected from Motoqua before (1990) during (1995–1996) and after (1999) a fire-associated population explosion (Fig. 2B). A similar lack of structure was found in the time series analysis from the DI Ranch (Fig. 2A) and Shivwits roadside washes (Fig. 2C). Similarly, the PCO did not yield any apparent population structure or site- or population- specific grouping associations.

### SET-II

**Figure 3:**
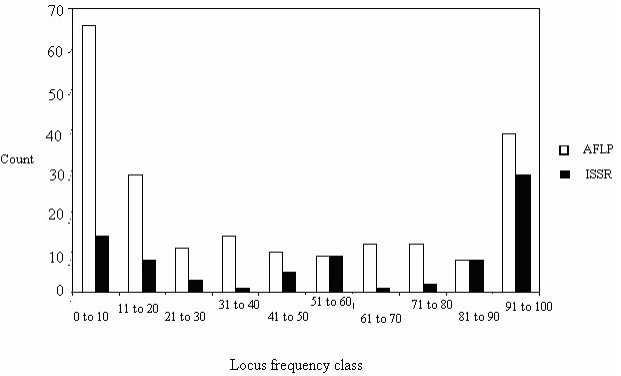
Locus frequency class distribution of 206 AFLP-(open) and 80 ISSR-(solid) loci from 175 ecotypes of *Nicotiana attenuata*.

Set II (Table 1) is a subset of Set I (Table 1) and consists of 175 individuals analyzed by AFLP and ISSR (either combined or separate) and this dataset was used for dendrogram and PCO analyses. Combined (AFLP + ISSR) analysis revealed a total of 286 loci of which 268 were polymorphic (93.70%). Here, the AFLP analysis showed higher percent polymorphic loci than did the ISSR analysis (96.1% and 87.5%, respectively; Table 2). Interestingly in the AFLP analysis, the primer and the restriction enzyme combinations that produced the lowest number of loci also delivered the highest rate of polymorphism (Table 2). It produced an average of 68.7 loci per primer combination with a high percentage of unique bands (65 in the 0–10% frequency class; Fig. 3) and a high frequency of commonly shared loci (42 in the 91–100% frequency class; Fig. 3). The ISSR analysis, on the other hand, produced 16 loci per primer with a predominance of commonly shared loci (29 in the 91–100% frequency class as compared to 14 in the 0–10% frequency class; Fig. 3). Dendrograms and PCO produced from this data set had the same overall characteristics as those produced from showed same structure nature Set I.

**Table 2:** AFLP and ISSR primers, total number of loci, polymorphic loci and percentage polymorphism.

**AFLP and ISSR Primer sequences (restriction enzymes)**	**Total loci**	**Nr. Polymorphic loci**	**% Polymorphism**
**AFLP**			
Eco-AGC\Mse-CAG	66	61	88.4
Eco-AAC\Mse-CCG	59	59	100.0
Eco-ACC\Mse-CCT	81	78	96.3
Total	206	198	96.1
**ISSR**			
(AG)_8_T	16	13	81.3
(GA)_8_T	13	11	84.6
(CT)_8_A	16	12	75.0
(CT)_8_G	26	25	96.2
(CA)_8_G	9	9	100.0
Total	80	70	87.5
Total of ISSR and AFLP	286	268	93.7

### Heterozygosity

A Bayesian approach [21] was used for heterozygosity calculations. The total heterozygosity as measured from the combined AFLP and ISSR data set of Utah collections (SET-II, Table 1: the 168 individuals from Utah without, Arizona California and Oregon) was 0.2771 ± 0.0018. The plants from Arizona California and Oregon were not included for heterozygosity and AMOVA analyses due to insufficient sampling of these populations. Different primer combinations produced different values; in particular, in the ISSR analysis, the (CA)_8_A primer produced comparatively high heterozygosity values. In contrast, the AFLP primers produced values that are more similar. Different regions had different measures of total heterozygosity with plants growing at the Lytle Ranch Preserve being the lowest (0.1881 ± 0.0052) and plants from Pahcoon, the highest (0.2043 ± 0.0027) (Table 3).

**Table 3:** Heterozygosity estimated using Bayesian approach within *N. attenuata *populations from different AFLP / ISSR primer combinations, total AFLP/ISSR heterozygosity and θ_B _estimates.

	Pahcoon	Rt 91	Motoqua	Lytle Ranch Preserve	Cedar Pockets	Jackson Spring	Shivwits Reserve	DI Ranch	Goldstrike Washes	Total	Fst (θ_B_)
**AFLP**											
E-AGC/M-CAG	0.1906 ± 0.0053	0.1975 ± 0.0079	0.2010 ± 0.0084	0.2135 ± 0.0091	0.2096 ± 0.0094	0.2233 ± 0.0090	0.1978 ± 0.0075	0.2055 ± 0.0103	0.2026 ± 0.0095	0.2152 ± 0.0044	0.0545 ± 0.0097
E-AAC/M-CCG	0.2224 ± 0.0059	0.2371 ± 0.0087	0.2334 ± 0.0094	0.2206 ± 0.0103	0.2363 ± 0.0103	0.2415 ± 0.0102	0.2176 ± 0.0087	0.2405 ± 0.0116	0.2269 ± 0.0109	0.2438 ± 0.0048	0.0598 ± 0.0095
E-ACC/M-CCT	0.2335 ± 0.0049	0.2370 ± 0.0071	0.2490 ± 0.0074	0.2531 ± 0.0083	0.2558 ± 0.0082	0.2453 ± 0.0082	0.2481 ± 0.0064	0.2530 ± 0.0092	0.2537 ± 0.0085	0.2606 ± 0.0041	0.0556 ± 0.0077
Total	0.2185 ± 0.0030	0.2260 ± 0.0044	0.2311 ± 0.0047	0.2332 ± 0.0052	0.2376 ± 0.0053	0.2387 ± 0.0053	0.2254 ± 0.0043	0.2357 ± 0.0058	0.2319 ± 0.0054	0.2432 ± 0.0024	0.0549 ± 0.0050
**ISSR**											
(AG)_8_GT	0.1969 ± 0.0139	0.1552 ± 0.0276	0.1644 ± 0.0277	0.1391 ± 0.0265	0.2211 ± 0.0237	0.2301 ± 0.0309	0.2384 ± 0.0156	0.1841 ± 0.0354	0.1252 ± 0.0301	0.2546 ± 0.0098	0.3250 ± 0.0591
(GA)_8_AT	0.1890 ± 0.0230	0.2007 ± 0.0285	0.1650 ± 0.0368	0.1554 ± 0.0372	0.1822 ± 0.0323	0.1597 ± 0.0389	0.2556 ± 0.0226	0.1669 ± 0.0421	0.1688 ± 0.0332	0.2517 ± 0.0179	0.3064 ± 0.0553
(CT)_8_A	0.3017 ± 0.0302	0.2816 ± 0.0345	0.3414 ± 0.0254	0.3659 ± 0.0258	0.3246 ± 0.0311	0.2701 ± 0.0346	0.2786 ± 0.0196	0.2850 ± 0.0393	0.2348 ± 0.0438	0.3600 ± 0.0167	0.1931 ± 0.0380
(CT)_8_G	0.2086 ± 0.0099	0.2172 ± 0.0139	0.2219 ± 0.0159	0.2135 ± 0.0160	0.2122 ± 0.0167	0.2220 ± 0.0157	0.2121 ± 0.0138	0.2235 ± 0.0181	0.2266 ± 0.0160	0.2284 ± 0.0099	0.0508 ± 0.0128
(CA)_8_G	0.2086 ± 0.0099	0.2172 ± 0.0139	0.2219 ± 0.0159	0.2135 ± 0.0160	0.2122 ± 0.0167	0.2220 ± 0.0157	0.2121 ± 0.0138	0.2235 ± 0.0181	0.2266 ± 0.0160	0.2284 ± 0.0099	0.1059 ± 0.0341
Total	0.2309 ± 0.0088	0.2190 ± 0.0100	0.2185 ± 0.0104	0.2145 ± 0.0109	0.2166 ± 0.0111	0.2188 ± 0.0118	0.2145 ± 0.0088	0.2190 ± 0.0131	0.2024 ± 0.0124	0.2452 ± 0.0056	0.1180 ± 0.0132
**AFLP + ISSR**											
**Total**	0.2043 ± 0.0027	0.1885 ± 0.0040	0.1916 ± 0.0044	0.1881 ± 0.0052	0.2001 ± 0.0053	0.2007 ± 0.0052	0.1919 ± 0.0036	0.1884 ± 0.0069	0.1844 ± 0.0056	0.2771 ± 0.0018	0.3305 ± 0.0088

### AMOVA

AMOVA analysis was performed separately for AFLP, ISSR and combined analysis of plants collected from Utah (168 individuals from SET-II, Tables 1, 4). The combined data set was also used to partition variation between wash and burn populations and to examine the effects of the collection year. In separate analyses, ISSR revealed higher variance than did the AFLP in the among-sites, among-population, and within-site categories; whereas, variation in the within-population category from the AFLP analysis was higher than that from the ISSR analysis (Table 4). All values except the among-site category in the AFLP analysis (*p *< 0.05) revealed highly significant differences at *p *< 0.001. AFLP and ISSR data was combined for an AMOVA analysis of all analyzed Utah populations. From this analysis, all three Φ categories were highly significant (*p *< 0.001; Table 4) among sites (Φct), among populations within sites (Φsc) and within populations (Φst) values were 0.046, 0.116, and 0.156, respectively. Table 4 reveals low genetic differentiation among sites and a relatively high genetic differentiation within populations. Pair-wise genetic distances (pair-wise Φst) were calculated from the AMOVA. Of the 300 comparisons from the 25 populations, 220 showed highly significant differences and 29 were significant at the p = 0.05 level (Table 5) [see Additional file 1]. Very low among-site variation (0.18 %) was obtained when samples were compared as being derived from either burn or wash populations (Table 4). To determine the effect of collection year, all individuals were grouped according to their collection year; an AMOVA analysis revealed low (3.77 %) variance within years at p < 0.5 significance level (Table 4).

**Table 4:** Summary of AMOVA analysis for 168 samples of *Nicotiana attenuata *individuals representing 25 populations from Utah region. Level of significance is based on 1000 iteration.

	Level of variation	df	Absolute	Percent	Φ values	*p*
Utah (AFLP+ISSR)	Among sites	8	1.38	4.59	Φct = 0.046	<0.001
	Among populations within sites	16	3.33	11.05	Φsc = 0.116	<0.001
	Within populations	143	25.44	84.36	Φst = 0.156	<0.001
AFLP	Among sites	8	0.59	2.72	Φct = 0.027	<0.05
	Among populations within sites	16	1.89	8.72	Φsc = 0.090	<0.001
	Within populations	143	19.17	88.55	Φst = 0.114	<0.001
ISSR	Among sites	8	0.79	9.38	Φct = 0.093	<0.001
	Among populations within sites	16	1.44	16.98	Φsc = 0.187	<0.001
	Within populations	143	6.27	73.68	Φst = 0.263	<0.001
Burn and wash	Among sites	1	0.05	0.18	Φct = 0.002	NS
	Among populations within sites	23	4.45	14.85	Φsc = 0.149	<0.001
	Within populations	143	25.44	84.97	Φst = 0.150	<0.001
Time	Among years	3	1.16	3.77	Φct = 0.038	<0.05
	Among populations within years	21	4.70	13.56	Φsc = 0.141	<0.001
	Within populations	143	25.44	82.66	Φst = 0.173	<0.001
Goldstrike	among populations	2	0.06	0.23	Φst = 0.002	NS
	within populations	5	25.86	99.77		
Motoqua	among populations	2	4.81	16.48	Φst = 0.165	<0.001
	within populations	12	24.39	83.52		
Pahcoon	among populations	8	3.19	12.06	Φst = 0.121	<0.001
	within populations	57	23.25	87.94		
Rt91	among populations	2	2.07	7.57	Φst = 0.076	<0.01
	within populations	18	25.27	92.43		
Shivwits	among populations	2	5.42	18.80	Φst = 0.188	<0.001
	within populations	21	23.39	81.20		

NS = Not significant

The AMOVA analysis had sufficient statistical power to detect small differences among populations, which accounted for 0.23 to 16.48 % of the variation, but this was dwarfed by the much larger genetic variation within populations, which ranged from 81.20 to 99.77 % (Table 4). This dramatic high degree of within-population variance was found in all populations. Again, populations from Goldstrike Canyon had the lowest among-population variance (0.23%) and highest within population variance (99.77%; Table 4). The Goldstrike populations were located in a narrow canyon produced by a stream and in this region seeds are likely transported among the populations during spring floods.

The Φ-statistic is an analogue of the F-statistic [22]. This analysis revealed that the Φct (i.e. among site variation) values were 0.046, 0.027 and 0.093 for the AFLP + ISSR, AFLP and ISSR analyses, respectively (Table 4). When these sites were grouped by burn and wash populations, very little genetic differentiation was observed (Φct 0.002). Interestingly, Φst (within population variation) was always comparatively high in all analyses (Table 4). Substantially higher Fst values were estimated by the program Hickory (Ver 1.0) [23] as θ_B _for the combined AFLP + ISSR analysis (0.3305 ± 0.0088) (Table 3), but surprisingly, the individual data set θ_B _values were lower (AFLP: 0.0549 ± 0.0050; ISSR :0.1180 ± 0.0132; Table 3).

Mantel test were conducted to analyze isolation by distance using pair-wise Φst values obtained by AMOVA (ver 1.55). The Φst values from the AFLP, ISSR and the combined data sets were separately correlated with geographical distance and all revealed non significant correlations (AFLP, r = 0.099, p = 0.81; ISSR, r = 0.122, p = 0.89 and AFLP + ISSR, r = 0.019, p = 0.63)

## Discussion

The analysis revealed high levels of heterozygosity, with total heterozygosity from all populations (0.2771 ± 0.0018) being higher than that from comparable analyses of self-pollinating annual plants (0.131) [24]. The ISSR analysis (0.2452 ± 0.0056) yielded estimates of heterozygosity that were comparable with the AFLP analysis (0.2432 ± 0.0024) (Table 3) despite the basic difference in the logic of the two procedures. ISSRs are designed to span a repeat region of the genome whereas AFLP is designed to randomly sample the full genome [16] and most plant genomes are thought to evolve faster in the repeat regions [25]. However, despite the differences in absolute estimates of genetic variation, both procedures produced the same conclusion.

The principle conclusion of this study is that large amount of genetic variation measured by AMOVA, within populations at a particular area significantly dwarfed that observed among sites, among populations growing in burns or washes, or collected during subsequent years growing at a given site. The conclusion that the genetic variation between neighbors is greater than that found between temporally- or spatially-separated populations, is dramatically reflected in the plants sampled along transects through the Pahcoon Springs burn. Only a small fraction (12.6%) of the total genetic variance is found among the 8 sub-samples from the extreme corners of this burn that covered more than 5000 hectares [26], while the majority (87.94 %) was found among plants growing within 10 m^2 ^of each other. Pahcoon, was the site from which highest number of populations were analyzed. (9 populations), whereas, from other sites smaller numbers of populations were analyzed.

F-statistic was estimated by using Bayesian approach with an analogue of the F-statistic, the Φ-statistic. Low but significant genetic differentiation was estimated among sites (Φct) from AFLP (0.027), ISSR (0.093) and AFLP + ISSR (0.046) data sets, whereas within populations, the Φst values were very high (Table 3). In *Platanthera leucophaea, *Holsinger *et. al *[21] showed that θ_B _is substantially larger than the estimates from the AMOVA analyses (θ_B _= 0.392 Vs Φst = 0,252). In our study, AFLP + ISSR analysis also showed such difference (θ_B _= 0.3305 Vs Φst = 0.156) whereas, exactly opposite values were found separate analysis of AFLP and ISSR data sets (AFLP. θ_B _0.0594 Vs Φst= 0.114; ISSR θ_B _= 0.1180 Vs Φst = 0.263).

A number of factors, including *N. attenuata*'s unusual seed germination mechanisms and the irregular nature of fires, natural selection, gene flow mediated by pollination or the relocation of seeds via mammal-vectored transport could account for the lack of population structure and each deserve further discussion.

Dormancy is a major adaptive response of native plants that allows them to cope with environmental variation and provide a means of habitat selection [27-29]. Moreover, dormancy is likely to influence the genetic structure of populations[30,31]. Seed banks serve as repositories of genetic diversity for most species. Many seeds use cues as general as temperature, photoperiod, moisture, or their own age to trigger germination and initiate vegetative growth [32,33]. To cope with the lack of reliability of these proximate signals and the unpredictability of the post-germination environment, some species may have evolved "bet-hedging" strategies, whereby only a certain fraction of the dormant seed bank germinates under favorable conditions. This has been experimentally shown by various researchers. In *Plantago lanceolata *[30], *Calluna vulgaris *[34], *Clarkia springvillensis *[35] and *Lesquerella fendleri *[36] it has been demonstrated that the seed banks have less genetic differentiation than do the adults of a given population. This strategy provides a statistical solution to the problem of cueing germination with unreliable signals [37,38]. Other species, however, use specific signals to time their germination with particular niches. Those species that specialize in the immediate post-fire habitat are a particular case in point [39]. Studies on another fire-dependant plant, *Grevillea macleayana *[40], which also has a long-lived seed bank, showed Fst (0.218) that were comparable to those measured in this study for *N. attenuata*, but had variable heterozygosity (H _obs _= 0.248 - 0.523). Another major difference from the current study was that *G. macleayana *showed significant isolation by distance.

Seed dormancy increases the effective generation time of this annual plant and by doing so, prevents genetic decay and inhibits the formation of spatial structure between geographically distinct populations [12]. Additionally, a long-lived seed bank results in the overlap of generations [41], which has similar effects and additionally reduces the ability of genetic drift to drive unique alleles to fixation. Operating under the assumption that the synchronized germination response observed after fires represents a synchronized germination of cohorts from the seed bank, we examined populations that occurred over a 6–11 year interval at the same location (Fig. 2A,2B,2C) to determine if temporally-defined genetic structure occurred in the populations, but none were found. This suggests that seed banks have a more complicated germination response whereby only fractions of a cohort may germinate at any particular interval and hence may represent a combination of "bet-hedging" [33] and the chemically-cued germination of the seed bank.

*N. attenuata *has all the characteristics of species pollinated by moths at night (white fragrant flowers scenting and becoming receptive at night) but day-active humming birds (*Selaphorus *sp.) and bumblebees (*Bombus *sp.) are also known to visit the flowers [42]. Despite these traits that are thought to facilitate out-crossing, 16 years of field work with the Utah populations have revealed that the vast majority of seeds produced are the result of self-pollination. No evidence exists for inbreeding depression in plants self-pollinated for more than 20 generations (I. T. Baldwin, unpublished results). However, the plant species likely enjoys sporadic bursts of cross pollination during the rare outbreaks of hawk moths (*Hyles lineata *and *Manduca *species(observed once in 16 years of observation at the study sites[43]. The amount and distance of gene flow that occurs during these rare events is not known. In the wind pollinated species such as *Zea mays *maximum distance of pollen dispersal was observed to be 18 m achieving outcrossing rate of not more than 1%; insect pollination does not substantially increase this rate [44]. Hence in comparison to seed dispersal these events are likely to have a minor effect on the homogeneity of populations [12].

Seeds of *N. attenuata *are small (160 μg) and could be dispersed by wind, water transport and animals, but none of these mechanisms are well documented. The seeds are eaten by various ground squirrels [45] but are not known to survive a transit through the digestive track. The greater heterogeneity within populations and low genetic differentiation among populations found along the stream in the Goldstrike canyon (Table 5) [see Additional file 1] suggests water transport may not be important. While seeds tend to be dispersed from the plant upon maturation of the seed capsules, the *N. attenuata *calyx is sticky and glandular and could be dispersed by adhering to animals. However, the plants ability to produce the defense secondary metabolite nicotine in substantial quantities in its calyx [9] may be a much more important determinant of its long distance transport. Native Americans are known to have smoked leaves and seed capsules for recreational and medicinal purposes, buried their dead with leather pouches containing *N. attenuata *seeds, burned the sagebrush to promote its growth and are likely to have transported seeds throughout its range in North America [46]. Hence, movement of *N. attenuata *genotypes across the landscape by humans who were smoking this plant may have contributed to the lack of correlation between geographical and pair wise Φst values, as reflected by Mantel test for isolation by distance.

In summary, we conclude that the unusual nature of the *N. attenuata *populations from Utah revealed by AFLP and ISSR analysis is a likely result of combination of random dispersal by humans and its seed dormancy.

## Conclusions

We conclude that the genetic structure of *N. attenuata *populations in Utah showed: 1) high similarity across collection sites; 2) small difference between populations growing in burns or washes; 3) small differences between growing seasons; and 4) large difference between individuals growing within populations.

## Methods

### Seed sources

Seeds were from individual- and multiple-plant samples collected from 1988–1999 from the southwestern USA (Table 1; Fig. 1). A majority of the seed collections (244) originated from a 1500 km^2 ^region of the SW corner of Utah (T38S R10W-T43S R19WUSA). Collections from Arizona (Flagstaff), Oregon (Eugene) and California (Sequoia Natl. Park) served as out-groups. In Utah, seeds were collected from plants growing at 6 locations for a number of years and were used for a time series analysis (Table 1). One of these areas (Motoqua), the region surrounding a small wash population that had been sampled in 1990, was struck by lightening at the end of the growing season in 1994 (August) and 1163 hectares were burned. During the 1995–6 growing seasons, large populations of more than 100,000 plants were found, but by 1999 only a small population remained in the original wash. At this site, seeds were collected during the population explosion as well during the contraction of the population at this site (Table 1). A fire during the 1998 growing season at Pahcoon Springs created a large population (covering more than 5,000 hectares) in the 1999 growing season which was sampled in 8 locations: seeds from 10 individual plants growing along each of 4 line-transects with an inter-plant distance of 10 m and 10 plants growing within a 10 m^2 ^area at 4 locations were sampled to provide a small-scale spatial analysis of genetic variation for this population.

### Plant material

Seeds (10 seeds per plant collected) were exposed for 1 h to 100 μL liquid smoke (House of Herbs INC., Passaic, NJ, USA): water (1:300, v/v) in 1-mL shell vials and 5 seedlings were planted in soil and grown to the rosette-stage in a glasshouse. Leaves from one plant randomly selected plant from each collection were harvested for DNA extraction.

### DNA extraction

Leaves were flash-frozen in liquid nitrogen, ground to powder and suspended in 750 μL of 100 mM Tris/50 mM EDTA (pH 8.0), containing 250 μg/mL RNase A. Eight μL liquid laundry detergent (Ariel, Procter & Gamble, Schwalbach, Germany) were added. After 60 min incubation at 60°C and subsequent addition of 80 μL of 5 M NaCl, the suspension was centrifuged for 5 min at 16,000 × g. The supernatant was removed and extracted with phenol/chloroform. The DNA was precipitated with 600 μL isopropanol, pelleted by centrifugation at 16,000 × g for 5 min, washed with 200 μL 70% ethanol and dissolved in 50 μL of water. The purity and concentration of the extracted DNA were assessed by electrophoresis on a 1% agarose gel and optical density spectrometric measurements. Both AFLP and ISSR procedures were performed on the same DNA samples.

### AFLP procedure

The four-step AFLP marker production procedure of Sharbel [47] was followed with minor modifications: (1) *Restriction*. The enzyme combination EcoRI/MseI was used to restrict 500 ng of genomic DNA per sample. A 10 μL digestion mix (*2 μL NEB Buffer #2, 2 μL 10 × BSA, 1.25 μL Mse I, 0.25 μL Eco RI, 4.5 μL water*) was added to 10 μL preparation of genomic DNA and incubated at 37°C for 3 h. (2) *Ligation of adaptators*. The double stranded EcoRI and MseI adaptator sequences designed by Zabeau and Vos [48] were ligated to the restriction fragments. A 40 μL ligation mix {6 μL ligase buffer (10 × Buffer for T4 DNA ligase)}, 4 pmol "EcoRI-adaptor", 2 pmol "Mse-adaptor", 0.6 μL T4 DNA ligase, water) was added to the 20 μL ligation reaction and incubated 19°C for 12 to 16 h. (3) *Pre-amplification*. Primers designed by Vos [14] complementary to a strand of each of the two adaptors with an additional selective nucleotide extension ("EcoRI-A" and "Mse-C") were used for an initial PCR pre-amplification step. An aliquot (17.5 μL) of a pre-amplification mix (2 μL 10 × Taq Buffer, 0.5 μL dNTP's (10 mM), 50 ng primer Mse-C (1μg/μL solution), 50 ng primer Eco-A (1μg/μL solution), water (grade III), and 0.1 μL Taq Polymerase B (5 U/μL)) was added to 2.5 μL of the digestion-ligation reaction in 0.2-mL PCR tubes. The PCR cycles are described in Sharbel [47]. (4) *Amplification*. Three 6-selective nucleotide EcoRI and MseI primer combinations, also designed by Vos [14] and demonstrated to be useful in *Nicotiana tabacum *by Ren & Timco [18], were used in the subsequent PCR amplifications. The primer combinations EcoRI-AGC/MseI-CAG; EcoRI-AAC/Mse-CCG; EcoRI-ACC/MseI-CCT were chosen with the help of successful studies by Ren & Timko [18]. Each of the three Eco RI-NNN primer types manufactured by Perkin-Elmer was labeled with a distinct fluorescent dye (either JOE, NED or HEX) at the 3' end to a ratio of 1:5. The following procedure was used for each of the three PCR reactions: 18 μL of amplification mix [2 μL 10 × Taq Puffer, 0.4 μL dNTP's (10 mM), 30 ng primer "EcoRI-NNN", 30 ng primer "Mse-NNN," water, and 0.1 μL Taq Polymerase B (5 U/μL)] was added to 2μL of the pre-amplification PCR product. PCR cycles were the same as in Sharbel [47].

The separate PCR amplification products generated by each of the three primer combinations were loaded together with a ROX 500 GeneScan size standard onto the ABI Prism 310 automated genetic analysis system as described in the manufacturer's instructions. The samples were run with the following GeneScan settings: "GS STR POP 4 (1 mL) D" module; 150000 V run Voltage; 5 second sample injection; 60°C gel temperature, and 9 m Watt's laser power. The distinct emission spectra of the three fluorescently labeled Eco RI-primer types made it possible to distinguish the DNA fragments resulting from each of the different primer combinations separately while the samples were being separated in the same electrophoresis capillary.

Collection of raw data and size alignment of the AFLP fragments was performed with ABI Prism GeneScan Analysis Software (Applied Biosystems) with the internal standard. Aligned data were subsequently imported into Genographer [49] for band calling. Each AFLP locus with an intensity ≥ 150 fluorescence units was scored with the 'thumbnail' option of genographer and converted into a 1/0 binary data matrix, which was used for further analysis.

### ISSR procedure

The PCR reaction (25 μL) contained 20 ng genomic DNA, buffer (10 mM Tris-HCl pH 8.3, 50 mM KCl, 1.5 mM MgCl_2_) 0.8 U *Taq *DNA polymerase (Eppendorf) 0.1 mM dNTPs, 0.3 μM primer. After 5 min initial denaturation at 94°C, 45 cycles of 1 min denaturation, 45 s annealing at 50°C, and 2 min for extension at 72°C, were followed by 5 min final extension in the PCR cycling program. A total of 55 primers were screened and 5 primers (Table 2) were selected because they reproducibly produced distinct banding patterns. The amplified products were separated on 2.0% agarose gel (28 samples plus 2-1 Kb ladder standards on each gel) in 0.5 × TAE buffer and bands were detected by ethidium bromide staining. The PCR reaction and separation of PCR products was carried out in duplicate for each DNA sample and only reproducible bands were scored manually as present (1) and absent (0).

### Data analysis

Pair-wise genetic similarity was calculated with the Jaccard coefficient [20]. The resulting matrix was processed for dendrogram construction using the UPGMA (unweighted pair group method average) clustering method and PCO (principle co-ordinate analysis) options of software MVSP (Multi-Variate Statistical Package ver 3.13: [50]) program. The entire AFLP (244 individuals. SET-I) and ISSR+AFLP (175 individuals, SET II) data sets were analyzed individually and the 175 individuals (Table 1) those were used in both procedures were combined for clustering analysis. Subsequently, the SET I data was analyzed for each time series separately (Fig. 2)

Genetic diversity was estimated for SET II (without Oregon, Arizona and California individuals) (168 individuals) as heterozygosity using the Bayesian approach of Holsinger et. al[21]. For this analysis, the analysis program, Hickory (ver 1.0) [23], was used with the full model. Several runs were carried out with default sampling parameters (burn-in = 50,000, sample = 250,000 and thin 50) to ensure consistency of results. Since, dominant markers (AFLP and ISSR) are used in conjunction with a largely sefling species, we used an approach that does not assume Hardy Weinberg equilibrium (Holsinger [21]).

The SET II was used to calculate molecular variance from the combined and separate AFLP and ISSR data sets (168 individuals) as partitioned into individual and population components with an AMOVA (ver1.55: [51]). We also calculated variation between different locations, burns and washes, by collection year and population, separately. Φ values generated by the AMOVA program were used to estimate pair-wise genetic diversity, which is an analogue of the F-statistic. The Mantel permutation test was used to correlate pair-wise Φst values obtained by separate analyses of the AFLP, ISSR and combined data sets with geographic distance.

## Authors' contributions

RB carried out the entire ISSR analysis, the analysis of the AFLP data and contributed to writing the manuscript. DS grew the plants, extracted the DNA and conducted the AFLP analysis. CAP collected seeds, grew the plants and extracted the DNA. ITB was responsible for coordinating the study, collecting seeds for the analysis, and wrote the manuscript.

## Supplementary Material

Additional File 1 Table 5 Pair-wise genetic difference (Φ st Lower diagonal of the matrix) among 25 populations of *Nicotiana attenuata*. Levels of significance are given in the upper diagonal of the matrix: *p < 0.05, **p < 0.01, ***p < 0.001 and NS, Non Significant at *p *< 0.05. *p*-value Indicates the probability that a random genetic distance (Φst) is larger than observed distance and are based on 1000 iterations steps. Click here for file
