# Supplementary material for: ISSR and AFLP analysis of the temporal and spatial population structure of the post-fire annual, Nicotiana attenuata, in SW Utah
Source: BMC Ecol. 2004 Sep 6;4:12. doi: 10.1186/1472-6785-4-12 (PMC517723; doi:10.1186/1472-6785-4-12)
Supplement: Additional File 1 — Table 5 Pair-wise genetic difference (Φ st Lower diagonal of the matrix) among 25 populations of Nicotiana attenuata. Levels of significance are given in the upper diagonal of the matrix: *p < 0.05, **p < 0.01, ***p < 0.001 and NS, Non Significant at p < 0.05. p-value Indicates the probability that a random genetic distance (Φst) is larger than observed distance and are based on 1000 iterations steps. [file 1472-6785-4-12-S1.doc]

**Table 5 Pair-wise genetic difference (Ф st Lower diagonal of the matrix) among 25 populations of *Nicotiana attenuata*. Levels of significance are given in the upper diagonal of the matrix: **p*<0.05, ***p*<0.01, ****p*<0.001 and NS, Non Significant at *p<*0.05. *p*-value Indicates the probability that a random genetic distance (Фst) is larger than observed distance and are based on 1000 iterations steps.**

| Region |  | Pahcoon | | | | | | | | | Rt91 | | |
| --- | --- | --- | --- | --- | --- | --- | --- | --- | --- | --- | --- | --- | --- |
|  | Code | P9 | P1 | P2 | P3 | P4 | P5 | P6 | P7 | P8 | R1 | R2 | R3 |
| Pahcoon | P9 |  | * | *** | * | * | *** | *** | *** | NS | *** | *** | *** |
|  | P1 | 0.05 |  | *** | *** | NS | *** | *** | *** | *** | *** | *** | *** |
|  | P2 | 0.08 | 0.11 |  | *** | NS | * | *** | *** | *** | *** | *** | *** |
|  | P3 | 0.06 | 0.10 | 0.10 |  | NS | *** | *** | *** | *** | *** | *** | *** |
|  | P4 | 0.05 | 0.07 | 0.03 | 0.06 |  | NS | *** | NS | * | * | *** | NS |
|  | P5 | 0.10 | 0.15 | 0.09 | 0.17 | 0.03 |  | *** | *** | * | *** | *** | *** |
|  | P6 | 0.18 | 0.26 | 0.20 | 0.25 | 0.19 | 0.18 |  | *** | * | *** | *** | *** |
|  | P7 | 0.10 | 0.09 | 0.10 | 0.09 | 0.06 | 0.15 | 0.22 |  | *** | *** | *** | *** |
|  | P8 | 0.07 | 0.13 | 0.09 | 0.12 | 0.06 | 0.07 | 0.05 | 0.06 |  | *** | *** | NS |
| Rt91 | R1 | 0.18 | 0.26 | 0.16 | 0.26 | 0.10 | 0.11 | 0.19 | 0.19 | 0.10 |  | NS | *** |
|  | R2 | 0.20 | 0.24 | 0.16 | 0.23 | 0.12 | 0.12 | 0.21 | 0.17 | 0.12 | -0.04 |  | *** |
|  | R3 | 0.12 | 0.17 | 0.15 | 0.15 | 0.06 | 0.08 | 0.16 | 0.10 | 0.08 | 0.12 | 0.12 |  |
| Motoqua | M4 | 0.14 | 0.23 | 0.15 | 0.22 | 0.10 | 0.11 | 0.18 | 0.20 | 0.14 | 0.14 | 0.16 | 0.11 |
|  | M5 | 0.23 | 0.40 | 0.29 | 0.38 | 0.24 | 0.20 | 0.11 | 0.35 | 0.15 | 0.25 | 0.28 | 0.20 |
|  | M6 | 0.21 | 0.34 | 0.20 | 0.32 | 0.14 | 0.19 | 0.22 | 0.28 | 0.17 | 0.10 | 0.14 | 0.21 |
| LPW | L1 | 0.08 | 0.13 | 0.11 | 0.18 | 0.09 | 0.09 | 0.18 | 0.16 | 0.10 | 0.18 | 0.19 | 0.14 |
| CP | C1 | 0.08 | 0.11 | 0.08 | 0.17 | 0.05 | 0.05 | 0.14 | 0.11 | 0.04 | 0.11 | 0.10 | 0.09 |
| JS | J1 | 0.07 | 0.07 | 0.05 | 0.13 | 0.02 | 0.04 | 0.16 | 0.09 | 0.03 | 0.09 | 0.11 | 0.07 |
| Shivwits | B7 | 0.24 | 0.30 | 0.21 | 0.29 | 0.16 | 0.18 | 0.25 | 0.25 | 0.14 | 0.18 | 0.19 | 0.22 |
|  | B5 | 0.18 | 0.27 | 0.22 | 0.28 | 0.12 | 0.16 | 0.26 | 0.21 | 0.16 | 0.18 | 0.19 | 0.20 |
|  | B8 | 0.20 | 0.21 | 0.24 | 0.30 | 0.21 | 0.23 | 0.24 | 0.21 | 0.17 | 0.22 | 0.20 | 0.25 |
| DI | D5 | 0.15 | 0.28 | 0.22 | 0.29 | 0.15 | 0.12 | 0.09 | 0.21 | 0.08 | 0.20 | 0.20 | 0.16 |
| Goldstrike | G1 | 0.12 | 0.22 | 0.18 | 0.24 | 0.08 | 0.09 | 0.17 | 0.18 | 0.10 | 0.14 | 0.17 | 0.12 |
|  | G2 | 0.17 | 0.26 | 0.16 | 0.26 | 0.05 | 0.12 | 0.15 | 0.23 | 0.10 | 0.16 | 0.16 | 0.14 |
|  | G3 | 0.07 | 0.27 | 0.22 | 0.18 | -0.02 | 0.07 | 0.04 | 0.16 | -0.02 | 0.14 | 0.17 | 0.02 |

Table 5 continuation

| Motoqua | | | LPW | CP | JS | Shivwits | | | DI | Goldstrike | | |
| --- | --- | --- | --- | --- | --- | --- | --- | --- | --- | --- | --- | --- |
| M4 | M5 | M6 | L1 | C1 | J1 | B7 | B5 | B8 | D5 | G1 | G2 | G3 |
| *** | * | *** | * | *** | * | *** | *** | *** | *** | * | *** | NS |
| *** | *** | *** | *** | *** | *** | *** | *** | * | NS | *** | *** | NS |
| *** | *** | *** | *** | *** | *** | *** | *** | *** | *** | *** | * | *** |
| *** | *** | *** | *** | *** | *** | *** | *** | *** | *** | *** | *** | *** |
| *** | *** | *** | *** | * | NS | *** | *** | *** | *** | * | ** | NS |
| *** | *** | *** | *** | NS | NS | *** | *** | *** | *** | *** | NS | NS |
| *** | NS | *** | *** | *** | *** | *** | *** | *** | NS | * | NS | NS |
| *** | *** | *** | *** | *** | *** | *** | *** | *** | *** | *** | *** | NS |
| *** | * | *** | * | * | * | *** | *** | *** | NS | *** | NS | NS |
| *** | *** | *** | *** | *** | *** | *** | *** | *** | *** | *** | NS | NS |
| *** | *** | *** | *** | * | * | *** | *** | *** | *** | *** | NS | NS |
| *** | * | *** | *** | *** | *** | *** | *** | *** | *** | *** | NS | NS |
|  | *** | *** | *** | * | *** | *** | *** | *** | *** | *** | *** | NS |
| 0.16 |  | *** | *** | * | *** | *** | *** | *** | NS | *** | *** | *** |
| 0.12 | 0.29 |  | *** | *** | * | *** | *** | *** | *** | *** | *** | *** |
| 0.12 | 0.21 | 0.16 |  | NS | NS | *** | *** | *** | NS | *** | *** | NS |
| 0.08 | 0.12 | 0.10 | 0.03 |  | NS | *** | *** | *** | * | *** | * | NS |
| 0.06 | 0.16 | 0.11 | 0.05 | 0.01 |  | *** | *** | *** | *** | *** | *** | NS |
| 0.19 | 0.25 | 0.22 | 0.21 | 0.12 | 0.11 |  | NS | *** | *** | *** | *** | NS |
| 0.15 | 0.25 | 0.19 | 0.18 | 0.10 | 0.08 | 0.05 |  | *** | *** | *** | *** | NS |
| 0.23 | 0.31 | 0.26 | 0.21 | 0.14 | 0.14 | 0.24 | 0.24 |  | *** | *** | *** | NS |
| 0.14 | 0.14 | 0.24 | 0.11 | 0.09 | 0.09 | 0.21 | 0.18 | 0.24 |  | * | NS | NS |
| 0.12 | 0.25 | 0.25 | 0.14 | 0.11 | 0.08 | 0.22 | 0.13 | 0.22 | 0.12 |  | NS | NS |
| 0.13 | 0.32 | 0.22 | 0.11 | 0.09 | 0.07 | 0.27 | 0.24 | 0.27 | 0.10 | 0.03 |  | NS |
| 0.00 | 0.28 | 0.29 | 0.08 | 0.07 | 0.00 | 0.28 | 0.23 | 0.24 | 0.02 | -0.02 | -0.19 |  |
